# Enteric nervous system damage caused by abnormal intestinal butyrate metabolism may lead to functional constipation

**DOI:** 10.3389/fmicb.2023.1117905

**Published:** 2023-05-09

**Authors:** Le Wang, Wan-Qiang Lv, Jun-Ting Yang, Xu Lin, Hui-Min Liu, Hang-Jing Tan, Ru-Ping Quan, Pan-Pan Long, Hui Shen, Jie Shen, Hong-Wen Deng, Hong-Mei Xiao

**Affiliations:** ^1^Center for System Biology, Data Sciences, and Reproductive Health, School of Basic Medical Science, Central South University, Changsha, Hunan, China; ^2^School of Basic Medical Science, Hunan University of Medicine, Huaihua, Hunan, China; ^3^Center of Safety Evaluation and Research, Hangzhou Medical College, Hangzhou, Zhejiang, China; ^4^Key Laboratory of Drug Safety Evaluation and Research of Zhejiang Province, Hangzhou Medical College, Hangzhou, Zhejiang, China; ^5^Department of Endocrinology and Metabolism, The Third Affiliated Hospital of Southern Medical University, Guangzhou, China; ^6^Tulane Center of Biomedical Informatics and Genomics, Deming Department of Medicine, Tulane University School of Medicine, New Orleans, LA, United States

**Keywords:** functional constipation, metagenomics, *Fusobacterium*, butyrate, enteric nervous system

## Abstract

Functional constipation (FC) is a high morbidity gastrointestinal disease for which dysfunction in the enteric nervous system is a major pathogenesis mechanism. To enhance our understanding of the involvement of intestinal microbiota and its metabolites in the pathogenesis of FC, we conducted a shotgun metagenomic sequencing analysis of gut microbiota and serum short-chain fatty acids (SCFAs) analysis in 460 Chinese women with different defecation frequencies. We observed that the abundance of*Fusobacterium_varium*, a butyric acid-producing bacterium, was positively correlated (*P* = 0.0096) with the frequency of defecation; however, the concentrations of serum butyric acid was negatively correlated (*P* = 3.51E-05) with defecation frequency. These results were verified in an independent cohort (6 patients with FC and 6 controls). To further study the effects of butyric acid on intestinal nerve cells, we treated mouse intestinal neurons *in vitro* with various concentrations of butyrate (0.1, 0.5, 1, and 2.5 mM). We found that intestinal neurons treated with 0.5 mM butyrate proliferated better than those in the other treatment groups, with significant differences in cell cycle and oxidative phosphorylation signal pathways. We suggest that the decreased butyrate production resulting from the reduced abundance of *Fusobacterium* in gut microbiota affects the proliferation of intestinal neurons and the energy supply of intestinal cells. However, with FC disease advancing, the consumption and excretion of butyric acid reduce, leading to its accumulation in the intestine. Moreover, the accumulation of an excessively high amount of butyric acid inhibits the proliferation of nerve cells and subsequently exacerbates the disease.

## Introduction

Functional constipation (FC) is one of the most common gastrointestinal diseases in clinical practice. The incidence rate of FC is approximately 14–17%, increasing with age (Bharucha et al., 2013; Bharucha and Wald, 2019). Until now, FC is believed to be related to low intestinal motility and poor lifestyle habits; however, its specific pathogenesis remains unclear (Aziz et al., 2020). The prevalence of FC in female patients is about 1.5 times higher than that in men, mainly because the drastic changes in hormone levels in women during menstruation and pregnancy affect the transportation of the small intestine and colon (Bharucha et al., 2013). Therefore, studying the pathogenesis of FC among female patients and identifying effective treatments to improve constipation is of great research value and social significance.

The enteric nervous system (ENS) plays a crucial role in the intestine. Their complex neural networks dominate and control intestinal function (Deb et al., 2020; Camilleri, 2021; Saldana-Morales et al., 2021; Vicentini et al., 2021). Previous studies have shown that the volume of colonic Cajal stromal cells in some patients with FC is significantly reduced, and the neuronal structure in the colonic circular smooth muscle layer is also reduced (He et al., 2000; Lyford et al., 2002; Tong et al., 2004), suggesting that the reduction of these cells may play an important role in the pathophysiology of chronic constipation. However, the specific mechanisms underlying neuronal decrease are unclear.

The human gut tract contains bacteria, archaea, viruses, and other microorganisms. Their metabolites include vitamins, short-chain fatty acids (SCFAs), secondary bile acids, choline metabolites, phenols, terpenoids, lipids, and hormones (Agus et al., 2021; Xiao et al., 2021). Normal microbiota and the external environment form a microecosystem that affects intestinal function through metabolites to resist external infections and improve autoimmunity.

Currently, SCFAs, secondary bile acids, and choline metabolites are considered to be the most important metabolites that regulate intestinal function. Because SCFAs can only be produced via glycolysis by anaerobic bacteria in the human intestine (Morrison and Preston, 2016), they are the most widely studied gut microbiota-derived metabolites. SCFAs are the main energy source for intestinal cells and are involved in various physiological functions within the host (Martin-Gallausiaux et al., 2021). In the ENS, SCFAs can regulate enteric neuronal survival and stimulate neurogenesis, but the specific mechanism is unclear (Vicentini et al., 2021).

Previous studies have shown that improving gut microbiota composition can alleviate FC. For example, common probiotics improve the intestinal microenvironment, enhance intestinal peristalsis and alleviate FC by increasing the concentration of 5-HT and SCFAs in the intestine (Miller et al., 2017; Martoni et al., 2019; Lewis et al., 2020; Wang et al., 2020). Fecal transplantation has also been used to treat patients with FC and achieved good efficacy (Yang et al., 2021; Zhang et al., 2021). However, our understanding of the involvement of gut microbiota and its metabolites in the pathophysiology of FC is still lacking.

Consequently, in this study, we performed a shotgun metagenomic sequencing analysis of gut microbiota and serum SCFAs analysis in 460 Chinese menopausal women with different defecation frequencies to explore the relationship between intestinal microbiota and their metabolites, intestinal peristalsis, and FC and subsequently validated the most significant findings in an independent sample. Furthermore, we performed *in vitro* cell experiments to verify the effect of butyrates on the proliferation of intestinal nerve cells.

## Results

### Information of 460 Chinese Han menopausal women

Overall, 518 perimenopausal and postmenopausal female volunteers were recruited in the Third Affiliated Hospital of Southern Medical University, Guangzhou, in November 2017, according to the inclusion and exclusion criteria in this study. We collected peripheral blood and fecal samples from these volunteers, performed metagenomic sequencing, and targeted the detection of serum SCFAs. After excluding individuals with missing data on one of the multi-omics data, we obtained multi-omics data from 460 Chinese Han menopausal women for the subsequent analysis. The participants were divided into the following four groups based on defecation frequency: group 1: >2 times/day, group 2: 1 time/day, group 3: 1 time/2 days, and group 4: <1 time/3 days. Referring to Rome IV for the diagnostic of FC, group 4 was identified as patients with FC, group 3 could be considered as high-risk people for FC, and groups 1 and 2 were low-risk people. There were no significant differences in age, menopausal age, and body mass index (BMI) among the four groups (Table 1 and Supplementary Table 1). However, since there were only 10 participants in group 4 and the data dispersion was high, we also combined groups 3 and 4 into one FC high-risk (FCHR) group for some of the subsequent analyses.

**TABLE 1:** Characteristics of study participants.

	Group 1	Group 2	Group 3	Group 4	FCHR	*P*
N	38	360	52	10	62	
Age Median (min–max), years	50.46 (44.16–54.75)	50.74 (40.06–61.2)	50.69 (44.39–54.98)	51.37 (48.87–53.98)	50.69 (44.39–54.98)	NS
Age at menopause Median (min–max), years	52.47 (46.4–57.95)	52.9 (41.47–63.8)	52.36 (46.32–56.61)	52.51 (50.13–56.02)	52.36 (46.32–56.61)	NS
BMI Median (min–max), kg/m^2^	24.64 (19.43–32.05)	22.22 (16.42–32.04)	22.25 (17.78–33.73)	24.15 (20.7–26.71)	22.25 (17.78–33.73)	NS

NS, not statistically significant, P > 0.05. The data were tested using Kruskal–Wallis test.

### Metagenomic sequencing revealed significant differences between FC low- and FC high-risk groups

**FIGURE 1:**
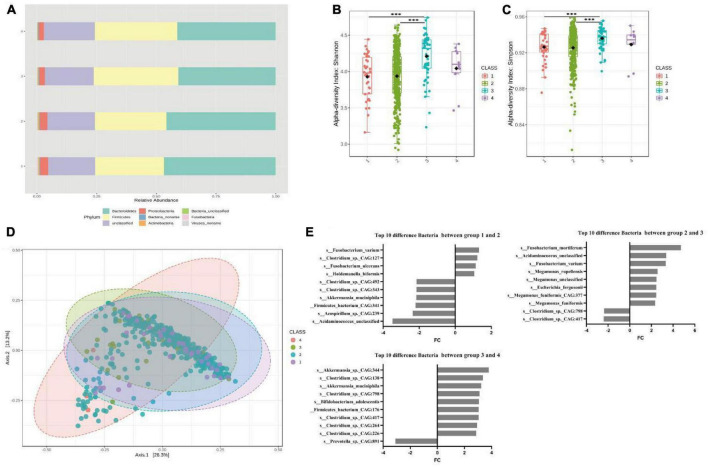
Significant bacterial structural differences between low- and high-risk groups of FC based on metagenomic data analysis. **(A)** The compositions and relative abundances of intestinal microorganisms in the four groups were different. **(B)** Shannon diversity in four groups. *P* = 1.00E-07 for the comparison between groups 1 and 3, *P* = 3.60E-05 for the comparison between groups 2 and 3. **(C)** Simpson diversity in four groups. *P* = 2.90E-05 for the comparison between groups 1 and 3, *P* = 7.95E-04 for the comparison between groups 2 and 3. **(D)** Principal coordinate analysis, *P* = 0.002 for the comparison between groups 1 and 3, *P* = 0.002 for the comparison between group 2 and 3. **(E)** The top 10 different bacteria species among the four groups obtained via Deseq2. In the comparison between groups 1 and 2, *Fusobacterium* (FC = 1.34, *P* = 0.0004). *s_Fusobacterium_varium* (FC = 1.32, *P* = 0.0038). *s_Akkermansia_muciniphila* (FC = –2.19, *P* = 2.62E-06), a positive FC indicates a decrease in relative abundance in the latter group of comparisons, and a negative FC indicates an increase in relative abundance in the latter group of comparisons. In the comparison between groups 2 and 3, *Fusobacterium* (FC = 2.64, *P* = 2.54E-17). *s_Fusobacterium_varium* (FC = 3.32, *P* = 5.61E-21). In the comparison between groups 3 and 4, *Acetobacter* (FC = 2.22, *P* = 6.52E-05). *Akkermansia* (FC = 2.05, *P* = 0.017). *s_Akkermansia_sp._CAG:344* (FC = 3.79, *P* = 6.67E-06), FC = log^2^ fold change, A positive FC indicates a decrease in relative abundance in the latter group compared between the two groups, and a negative FC indicates an increase in relative abundance in the latter group compared between the two groups.

We performed gut microbiota profiling in the four groups through shotgun metagenomic sequencing. After data quality control and filtering, the average number of microbes obtained from each sample was 10,720. For subsequent analyses, we focused on 625 bacterial species whose relative abundance was greater than 0.01% (Supplementary Table 2). As shown in Figure 1A, at the phylum level, groups 1 and 2 had higher abundances of *Bacteroides*, Fusobacteria, and Proteobacteria than groups 3 and 4, while groups 3 and 4 had a higher abundance of Firmicutes. Similarly, at the genus level, groups 1 and 2 had higher *Bacteroidaceae_ Bacteroides* and lower Firmicutes levels than groups 3 and 4 (Supplementary Figure 1). These results indicated that there were significant differences in the composition of gut microbiota among people with different defecation frequencies. Moreover, we conducted α and β diversity analyses of intestinal microorganisms and observed that there were differences in biodiversity among the four groups. Shannon and Simpson indices and the principal coordinates analysis (PCoA) based on Bray Curtis distances showed that the biodiversity of groups 1 and 2 was lower than that of groups 3 and 4, and there were statistically significant differences between groups 1 and 3, as well as groups 2 and 3 (Figures 1B–D).

To clarify the correlation between the type of bacteria and defecation frequency, we used DESeq2 to compare the bacterial operational taxonomic units (OTUs) among the four groups and obtained the top 10 bacteria with the largest fold change. The results showed that *Fusobacterium* was the differential bacteria with the greatest fold change in groups 1 and 2, as well as groups 2 and 3, while *s_Fusobacterium_varium* was the only bacteria that coexisted in the two differential lists. In groups 1 and 2, we also found an increase in the relative abundance of *s_Akkermansia_muciniphila*, which may lead to intestinal damage. In groups 3 and 4, we found that *Acetobacter* and *Akkermansia* were the top two differential bacteria with a decrease in relative abundance, among which *s_Akkermansia_sp._CAG:344* was the bacteria with the largest fold change (Figure 1E and Supplementary Table 4). Therefore, comparing the FC low- and high-risk groups, we observed that *Fusobacterium*, a butyric acid-producing bacterium, was the key differential bacterium in groups 1, 2, and 3 (Supplementary Figure 3). The abundances of *s_Fusobacterium_varium* and *s_Fusobacterium_mortiferum* in the population were positively correlated with the frequency of defecation (Supplementary Figure 4). In contrast, we found no significant difference in other butyrate-producing bacteria and methanogens such as *Faecalibacterium_prausnitzii*, *butyrateimonas_virosa*, *Lactobacillus*, *Bifidobacterium*, and *methanogenic* in the four groups (Supplementary Table 6). Similar results were also obtained when comparing the FCHR group with groups 1 and 2 (Supplementary Figure 5).

### Butyric acid was significantly different among the four groups

**FIGURE 2:**
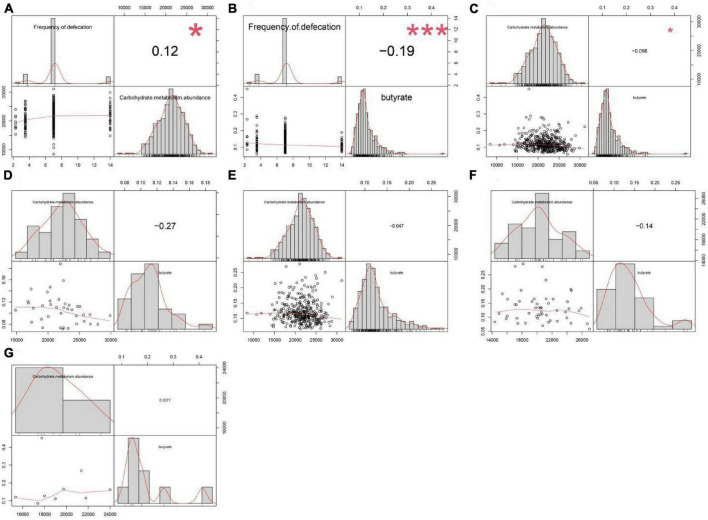
Correlation analysis between the abundance of carbohydrate metabolism pathway, defecation frequency, and serum butyric acid concentration. The abundance of carbohydrate metabolism pathway in gut microbiota was **(A)** positively correlated (*P* = 0.013) with defecation frequency but **(C)** negatively correlated (*P* = 0.036) with serum butyric acid levels across the four groups. **(B)** Defecation frequency was negatively correlated (*P* = 3.51E-05) with serum butyric acid levels across the four groups. There was no significant correlation between the abundance of carbohydrate metabolism pathway and serum butyric acid concentration in **(D)** group 1 (*P* = 0.105), **(E)** group 2 (*P* = 0.373), **(F)** group 3 (*P* = 0.314), and **(G)** group 4 (*P* = 0.984).

We compared the serum SCFA levels among the four FC groups and found that butyric acid was significantly different between groups 1 and 2, 1 and 3, and 1 or 2 and the FCHR group (Table 2). In addition, serum butyric acid levels were negatively correlated with the frequency of defecation (Figure 2B). These results suggested that serum SCFAs concentration, especially butyric acid, may be closely related to intestinal defecation function.

**TABLE 2:** SCFAs concentration in the four FC groups.

	Group 1	Group 2	Group 3	Group 4	FCHR	*P*
*N*	38	361	52	10	62	
Butyric acid Median (95% CI)	0.1067 (0.0978–0.1146)	0.1129 (0.1178–0.1254)	0.1234 (0.1199–0.1491)	0.1236 (0.0859–0.2492)	0.1234 (0.1248–0.1581)	a, b, c, d < 0.05
Valeric acid Median (95% CI)	0.036 (0.0316–0.0468)	0.0369 (0.0391–0.0432)	0.0394 (0.0385–0.0506)	0.0433 (0.0267–0.0908)	0.0394 (0.04–0.0536)	NS
Acetic acid Median (95% CI)	0.4792 (0.4717–0.6958)	0.5566 (0.5899–0.6622)	0.6434 (0.5974–0.7998)	0.5265 (0.3904–0.7464)	0.6434 (0.5889–0.7663)	NS
Caproic acid Median (95% CI)	0.0434 (0.041–0.0488)	0.0493 (0.0494–0.0527)	0.0486 (0.0462–0.0596)	0.0538 (0.0391–0.0632)	0.0486 (0.0468–0.0584)	a = 0.034

Data are expressed as the median (95% confidence interval, 95% CI). The data were analyzed using Kruskal–Wallis test. a: P = 0.012 for the comparison between group 1 and group 2. b: P = 0.01 for the comparison between group 1 and group 3. c: P = 0.031 for the comparison between group 1 and group FCHR. d: P = 0.003 for the comparison between group 2 and group FCHR. Caproic acid: a, P = 0.034. NS, not statistically significant.

### Functional analysis of metagenomic sequencing revealed a significantly reduced capacity to metabolize carbohydrates in FC high-risk groups

We further explored the potential differences of bacterial metabolic pathways in different groups to identify gut microbiota functional capacity associated with FC. After quality control and host gene removal, clean reads were mapped to 1,048,576 unigenes in the integrated gene catalog (Homo_sapiens.GRCh38.dna_sm.chromosome.chr.fa). The differential metabolic pathway analysis [based on Kyoto Encyclopedia of Genes and Genomes (KEGG) level 2 modules] detected eight differential metabolic pathways between groups 2 and 3 (Table 3), and similar results were obtained when comparing the FCHR group with groups 1 and 2 (Supplementary Table 7). In group 3 and the FCHR group, the abundance of metabolic pathways for cofactors and vitamins metabolism, xenobiotics biodegradation and metabolism, and carbohydrate metabolism decreased, whereas pathways for translation, cell growth and death, and replication and repair increased (Table 3 and Supplementary Table 7). These differential metabolic pathways suggested that with an advancing degree of FC disease, the metabolic ability of intestinal microbes to utilize and metabolize exogenous substances decreased; however, the rate of the renewal of microbes increased.

**TABLE 3:** Significant differential gut microbial metabolic pathways between groups 2 and 3.

KEGG pathways (level 2)	Abundance (group 2)	Abundance (group 3)	*P*	FDR
Carbohydrate metabolism	21,606.61	20,442.84	0.015	0.09
Metabolism of cofactors and vitamins	11,610.47	11,154.69	0.040	0.13
Xenobiotics biodegradation and metabolism	610.51	571.78	0.035	0.15
Cell growth and death	729.52	775.5	0.012	0.16
Infectious diseases	936.58	999.27	0.021	0.11
Replication and repair	6,732.43	7,031.62	0.014	0.12
Translation	9,482.93	10,140.87	0.007	0.18
Membrane transport	14,836.23	15,656.07	0.035	0.13

Carbohydrate metabolism was closely associated with butyric acid metabolism, and the correlation analysis across the four groups showed that the frequency of defecation was positively correlated with the abundance of carbohydrate metabolism pathway, but carbohydrate metabolism was negatively correlated to the concentration of serum butyric acid (Figures 2A, C). However, there was no significant correlation between serum butyric acid concentration and the abundance of carbohydrate metabolism pathways within each individual group (Figures 2D–G). These results showed that butyric acid production capacity directly affects the concentration of butyric acid absorbed into the blood in the intestine, potentially affecting the frequency of defecation.

### The results of metagenomic and SCFAs analysis in the validation population were consistent with those in the experimental discovery population

**FIGURE 3:**
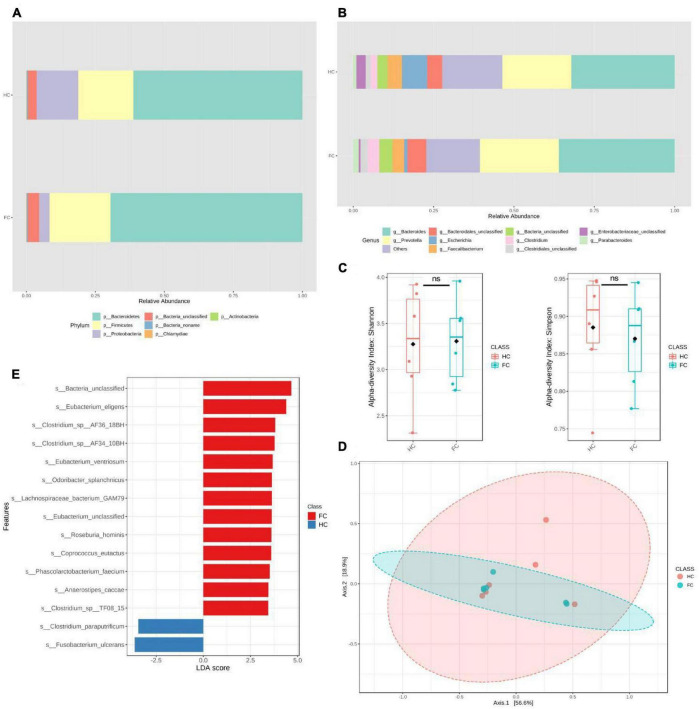
Significant bacterial structural differences between HC and FC groups were found based on metagenomic data analysis. **(A,B)** The relative abundance of intestinal microbiota in phyla and genus levels of the two groups. **(C)** Alpha diversity analysis, Simpson, *P* = 0.52, Shannon, *P* = 0.87. **(D)** PCoA, *P* = 0.71. **(E)** The LDA distribution diagram analysis showed a clear alteration of the microbiota characterized by higher *s_Fusobacterium ulcerans* levels in the HC group (LDA score >2.5). In the comparison between groups HC and FC, we found *g_Fusobacterium* (LDAscore = –3.74, *P* = 0.025), *s_Fusobacterium_ulcerans* (LDAscore = –3.64, *P* = 0.037), *s_Fusobacterium_mortiferum* (LDAscore = –3.05, *P* = 0.025), *s_Lachnospiraceae_bacterium_GAM79* (LDAscore = 3.64, *P* = 0.038), *s_Bacteroides_sp_4_1_36* (LDAscore = 3.25, *P* = 0.025), *s_Bacteroides_fluxus* (LDAscore = 3.11, *P* = 0.01), *s_Bacteroides_sp_ AM54_2NS* (LDAscore = 2.82, *P* = 0.01). A positive LDAscore indicates a decrease in relative abundance in the HC group, and a negative LDAscore indicates an increase in relative abundance in the HC group. LDAscore, linear discriminant analysis score. A positive LDAscore indicates an increase in relative abundance in the FC group compared with the HC group, and a negative LDAscore indicates a decrease in relative abundance in the FC group compared with the HC group.

To further validate our findings, we recruited an independent sample of 12 premenopausal women, including 6 patients with FC and 6 healthy controls (HC), in Huai Hua City, Hunan Province, China. There were no significant differences in age and BMI between the FC and HC groups (Table 4). Metagenomics next-generation sequencing (mNGS) and targeted SCFAs were used to evaluate fecal and serum samples. MicrobiomeAnalyst (Chong et al., 2020) was used for analysis after obtaining the filtered data. We compared the structural composition of the intestinal microbiota of 460 people with that of the 12 people. The results showed that there were differences in the structure of intestinal microbiota between the two groups (Figures 3A, B). At the gate level, although the structure of the intestinal microbiota of the two groups was similar, there were significant differences. Among 460 people, the top five in relative abundance were *Bacteroides*, *Clostridium*, *Eubacterium*, Firmicutes, and Roseburia, while among the 12 people were *Bacteroides*, *Prevotella*, *Escherichia*, *Faecalibacterium*. Moreover, the Simpson and Shannon indices of the 12 people were significantly lower than that of the 460 people. This difference in intestinal microbiota structure was attributed to geographic differences (He et al., 2018).

**TABLE 4:** Characteristics of the independent replication cohort.

	FC	HC	*P*
***N***	6	6	
Age Median (min–max), years	47.5 (43–51)	46.5 (42–50)	NS
BMI Median (min–max), years	22.21 (19.88–29.63)	21.53 (19.72–25.91)	NS

NS, not statistically significant. P > 0.05. The data were tested by Kruskal–Wallis test.

The validation population analysis results showed that the Shannon and Simpson indices and PCoA were not significantly different between the two groups (Figures 3D, E). The results of differential intestinal microbes obtained by linear discriminant analysis (LDA) showed that at the genus level, only the relative abundance of *g_Fusobacterium* decreased in the FC group compared with the HC group (LDAscore = −3.74, *P* = 0.025). At the species level, *s_Fusobacterium_ulcerans* was the most significantly reduced bacteria (LDAscore = −3.64, *P* = 0.037), and *s_Fusobacterium_mortiferum* (LDAscore = −3.05, *P* = 0.025) was also at the top. Several other species also showed higher relative abundance in the HC group, including *s_Lachnospiraceae_bacterium_GAM79*, *s_Bacteroides_sp_4_1_36*, *s_Bacteroides_fluxus*, and *s_Bacteroides_sp_AM54_2NS* (Figure 3E and Supplementary Table 5). Among the tested serum SCFAs, only butyric acid showed a significant difference (*P* = 0.002) between the FC and HC groups (Table 5).

**TABLE 5:** SCFAs concentration in the replicating cohort.

	HC	FC	*P*
*N*	6	6	
Butyric acid Median (95% CI)	0.018 (0.014–0.021)	0.033 (0.016–0.068)	0.002
Valeric acid Median (95% CI)	0.0109 (0.01–0.012)	0.627 (0.46–0.82)	NS
Acetic acid Median (95% CI)	0.5235 (0.44–0.58)	0.627 (0.43–0.83)	NS
Caproic acid Median (95% CI)	0.0311 (0.027–0.035)	0.0341 (0.029–0.042)	NS

Data are expressed as the median (95% confidence interval, 95% CI). The data were analyzed using Kruskal–Wallis test. NS, not statistically significant, P > 0.05.

Overall, we obtained similar results by analyzing two groups of women with different living places and ages; the abundance of *Fusobacterium* in the intestine was positively correlated with the frequency of defecation; however, serum butyric acid was the opposite.

### Intestinal nerve cells can only develop well in an appropriate concentration of butyrate

**FIGURE 4:**
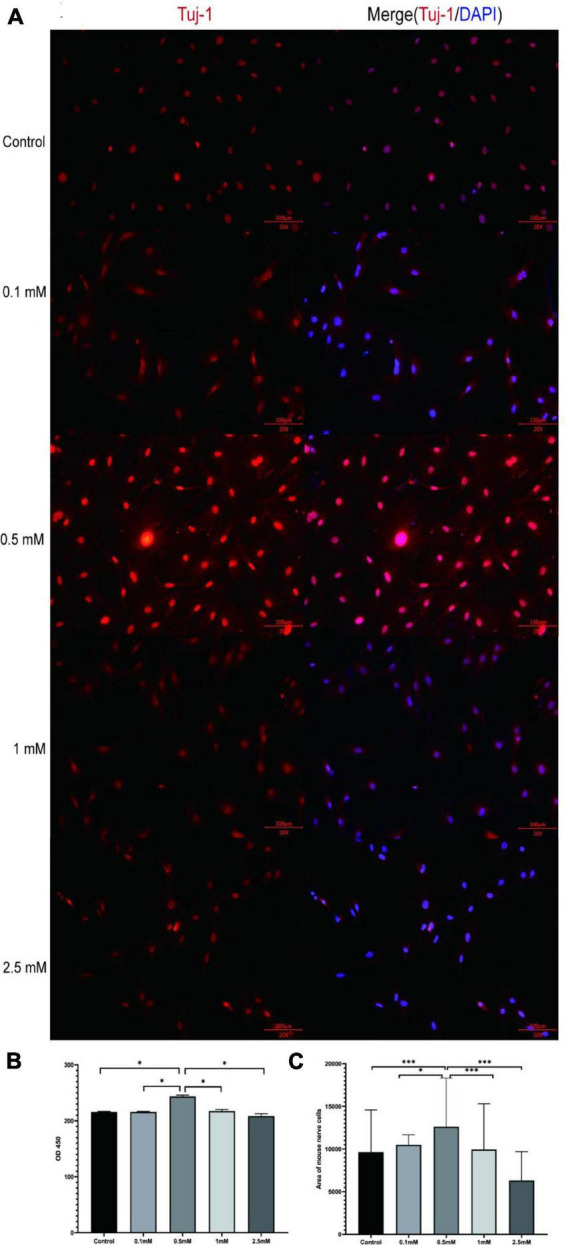
Butyrate affects intestinal nerve cell proliferation *in vitro*. **(A)** The nerve cells were cultured with different concentrations of butyrate (0, 0.1, 0.5, 1, and 2.5 mM) for 72 h and stained with nerve-specific antibody Tuj1 and DAPI. **(B,C)** Cells cultured in 0.5 mM butyrate showed significantly higher cell proliferation rate (assessed by OD450) and area coverage than cells cultured under all the other conditions. *P < 0.05, ***P < 0.001.

Butyrate plays an important role in intestinal energy supply, nerve signal transduction, and maintenance of intestinal homeostasis (Soret et al., 2010; Su et al., 2021). To study whether butyrate affects the proliferation of intestinal nerve cells, we isolated intestinal nerve cells from C57BL/6 mice, and according to a previous study (Su et al., 2021), we chose five different butyric acid concentrations (0, 0.1, 0.5, 1, and 2.5 mM) to culture the cells for 72 h (Figure 4 and Supplementary Figure 2). We found that the nerve cells cultured in 0.5 mM butyrate proliferated significantly faster and showed a better growth state than those cultured under other conditions (Figures 4B, C). These results demonstrated that the proliferation of intestinal nerve cells was directly affected by butyrate concentration.

### Butyrate regulates the proliferation of intestinal nerve cells through the cell cycle and oxidative phosphorylation signaling pathways

**FIGURE 5:**
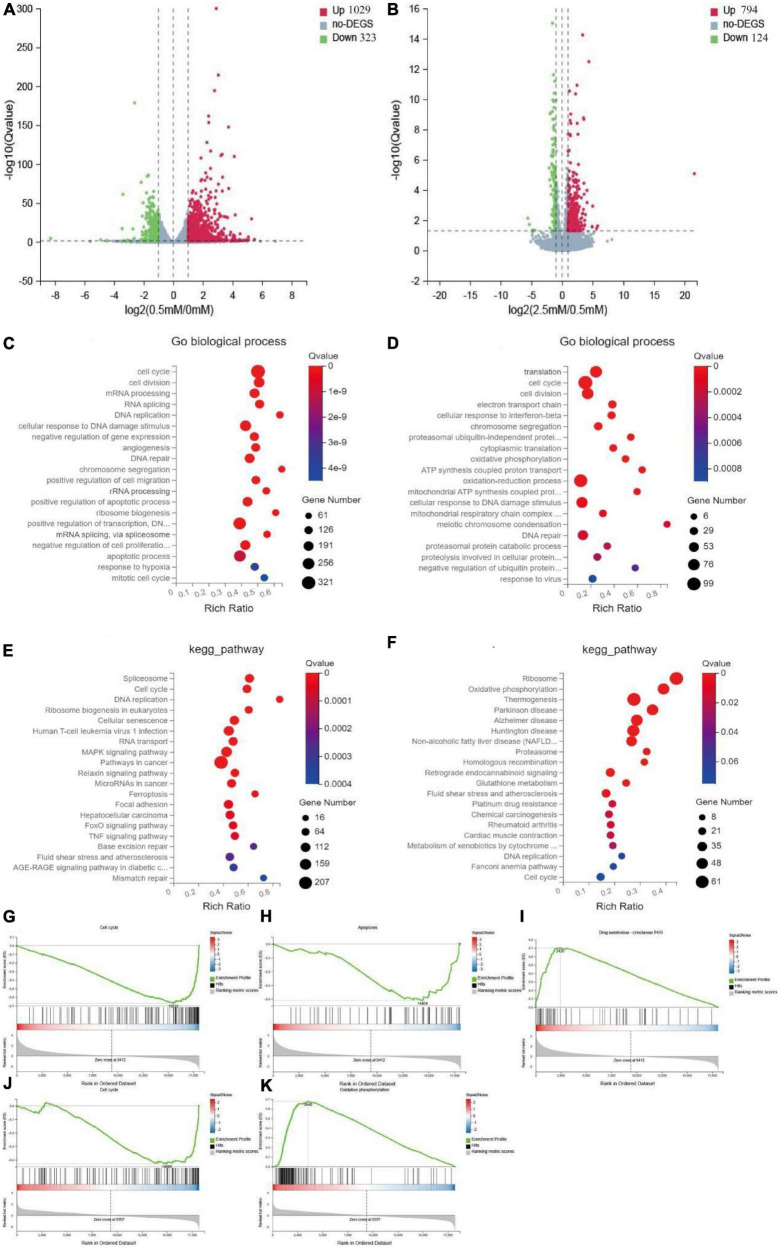
Mechanism of butyrate on the proliferation of intestinal nerve cells. **(A,B)** Volcano plots show up-regulated and down-regulated genes of 0.5 mM/0 mM and 2.5 mM/0.5 mM, fold change > 1. **(C,E)** Top 20 of the functional and pathway analysis by go biological process and KEGG of 0.5 mM/0 mM. **(D,F)** Top 20 of the function and pathway analysis by go biological process and KEGG of 2.5 mM/0.5 mM. **(G–I)** Top-ranked signaling pathways related to cell proliferation in GSEA analysis of 0.5 mM/0 mM comparison group. Cell cycle and apoptosis were up-regulated, and the metabolism of xenobiology by cytochrome P450 was downregulated. **(J,K)** Top-ranked signaling pathways related to cell proliferation in GSEA analysis of 2.5 mM/0.5 mM comparison group. Oxidative phosphorylation was up-regulated, and the cell cycle was down-regulated.

To further investigate the specific mechanism underlying the observed differences in intestinal nerve cell proliferation under different butyrate concentrations, we performed RNA-seq on mouse intestinal nerve cells after culturing them with various concentrations of butyrate (0, 0.5, and 2.5 mM) for 72 h. Compared with the 0.5 mM group, 1,029 genes were significantly up-regulated and 323 genes were down-regulated in the 0 mM group; whereas, compared with the 2.5 mM group, 794 genes were up-regulated and 124 genes were down-regulated in Figures 5A, B.

We performed gene ontology (GO), KEGG, and Gene Set Enrichment Analysis (GSEA) enrichment analyses on these differentially expressed genes (DEGs). Compared with the 0.5 mM group, the GO biological process enriched in the 0 mM group was related to cell cycle, DNA repair, and apoptotic process (Figure 5C), while the biological process enriched in the 2.5 mM group was related to cell cycle, cellular response to DNA damage stimulus, and oxidative phosphorylation (Figure 5D). KEGG and GSEA analyses showed that 21 pathways were up-regulated and 48 pathways were down-regulated in 0 mM group. Among them, cell cycle, DNA replication, and apoptosis were up-regulated, and cytochrome P450 was down-regulated in xenobiology (Figures 5E, G–I) and Supplementary Table 7). Forty-one pathways were up-regulated, and four pathways were down-regulated in the 2.5 mM group. Among them, oxidative phosphorylation, glutathione metabolism, and NAFLD were up-regulated, and cell cycle and regenerative recombination were down-regulated (Figures 5F, J, K and Supplementary Table 8). These are key pathways closely related to cell proliferation.

Therefore, although lack of butyrate and high concentration of butyrate inhibited the proliferation of nerve cells, they were caused by different mechanisms. The possible mechanism of lack of butyrate may be cell cycle disorder and subsequent apoptosis, while the high concentration of butyrate may be a series of cell damage and cell cycle inhibition caused by increased oxidative phosphorylation.

## Discussion

By studying female intestinal microbes and their metabolites and *in vitro* evaluation of butyrate effects on nerve cells, we believe that the reduction of *Fusobacterium* may be one of the causes of constipation. The experiment showed that butyrate with proper concentration could provide a better environment for the proliferation of nerve cells. Therefore, the maintenance of intestinal butyrate concentration is essential for intestinal function.

Intestinal microbes are closely associated with intestinal functions. Many studies have shown that there are many butyrate-producing bacteria in the gut, including *F. prausnitzii* and *B. virosa* (Thompson et al., 1992; Hertel et al., 2021). In this study, the abundant distribution of *F. prausnitzii* and *B. virosa* in the whole study population was not different; hence, *Fusobacterium* may play a more vital role than those two butyrate-producing bacteria in patients with FC.

In this study, we observed that the diversity of intestinal microbes was negatively correlated with the frequency of defecation. This result is consistent with the finding from a recent study where gut microbiota diversity and species of *Prevotella* and *Bacteroides* were significantly decreased in patients with chronic constipation (Dan et al., 2020). Constipation destroys the intestinal microenvironment, reduces the abundance of dominant microorganisms such as *Prevotella* and *Bacteroides*, and increases the less abundant harmful microorganisms, such as *Escherichia* and Enterobacteriaceae, thus leading to increased gut microbial diversity.

Firmicutes and Bacteroidetes are the core microbiota of the human gut, both of which participate in many physiological activities. Previous studies have suggested that the relative abundance ratio of Firmicutes/Bacteroidetes (F/B) in adults should be maintained within a normal range because either too high or too low has been associated with the diseases (Magne et al., 2020). Jeffery et al. (2020) reported that the F/B value could differentiate subtypes of patients with irritable bowel syndrome (IBS) in clinical practice (Stojanov et al., 2020). Liu et al. (2018) found that the F/B value of patients with liver cirrhosis was higher than that of the healthy control group. Moreover, we found that in the four groups of samples, F/B gradually increased with the increase in population FC risk, indicating the potential of abnormal F/B values as a clinical warning indicator for FC. *Prevotella* is also a dominant bacterium in the gut. Recent studies have reported that *Prevotella* can utilize polysaccharides to produce succinic acid, which enhances the immune response of antigen-specific T cells and protects host health by binding to the succinic acid receptor GPR91 on the surface of dendritic cells (Rubic et al., 2008). Additionally, inflammation caused by immune deficiency is one of the most common symptoms in FC (Rosa et al., 2023); consequently, we speculate that this is related to a decrease in the relative abundance of *Prevotella*.

This study showed a seemingly paradoxical result—the abundance of *Fusobacterium*, a butyrate-producing bacterium in the intestine, was decreased in patients with FC, whereas the serum butyric acid concentration was increased in patients with FC. In addition, the blood concentrations of other SCFAs, such as valeric acid, acetic acid, and caproic acid, were also increased (though not statistically significant) with the decrease in defecation frequency. However, similar results have been reported in studies for FC and Parkinson’s disease (PD, approximately 40–80% of patients with PD exhibit constipation or prolonged colonic transit) (Shin et al., 2020; Tian et al., 2021; Chen et al., 2022; Yang et al., 2022). For instance, Tian et al. (2021) observed a relative decrease in SCFA-producing bacteria in intestinal microbiota in patients with constipation, and Yang et al. (2022) found that patients with PD and constipation had lower levels of SCFAs in fecal samples but higher SCFA levels in plasma than in that of patients with PD without constipation. A possible explanation is that the reduction of intestinal peristalsis and defecation frequency in patients with constipation leads to decreased SCFAs consumption and excretion in the gut, which in turn results in more SCFAs are accumulated in the intestine and absorbed into the blood, particularly with an increased intestinal permeability (Tian et al., 2021).

Nerve cells play important roles in the intestine. The ENS in most areas of the intestine comprises two main ganglion layers as follows: the intermuscular and submucosal ganglions, which contain many types of intestinal neurons and glial cells. Axons from the ENS and extrinsic neurons innervate most layers of the intestinal wall and regulate several intestinal functions. One of the main pathogenesis mechanisms of FC is the dysfunction of the colonic smooth muscle nerve, which leads to slow intestinal peristalsis and causes disease (Tong et al., 2004; Deb et al., 2020; Camilleri, 2021; Saldana-Morales et al., 2021). Studies have confirmed that SCFAs play a crucial role in the ENS, including providing energy for the ENS and participating in regulating colon transport by controlling the increase of 5-HT concentration (Lin et al., 2021). However, there is no study on the relationship between SCFAs and the proliferation of the intestinal nervous system. Our research shows that an appropriate concentration of butyrate can effectively promote the proliferation of mouse intestinal nerve cells. Both low and high concentrations of butyrate can affect the normal cell cycle and damage the repair of intestinal nerve cells, possibly through different mechanisms.

One of the major pathways down-regulated in nerve cells by the lack of butyrate is the metabolism of xenobiology by cytochrome P450. This pathway is involved in regulating various cell signal transduction pathways and normal homeostasis that are crucial to the cell cycle (Navarro-Mabarak et al., 2018; Wang and Yao, 2021). When the CYP450 pathway is out of balance, the cycle of nerve cells will be disrupted, and the expression of cell cycle-related pathways and apoptosis-related pathways will be up-regulated, eventually leading to apoptosis (Wang and Yao, 2021). Another vital protein is the aryl hydrocarbon receptor (AHR), which can bind with various ligands to activate intracellular signals. Some studies have reported that a reduction of *AHR* expression will lead to a slowdown of intestinal peristalsis (Zapletal et al., 2017; Obata et al., 2020), while the expression of AHR in the 0 mM group decreased significantly (Supplementary Table 11). This finding may be the main factor leading to slow intestinal peristalsis in patients with FC.

Excessively high butyrate concentration mainly leads to the up-regulation of the neuronal oxidative phosphorylation pathway and down-regulation of cell cycle and endogenous recombination pathways. The increased activity of the oxidative phosphorylation pathway will lead to the increase of reactive oxygen species (ROS) and neuronal apoptosis rate (Rose et al., 2018; Singh et al., 2019). It has been reported that a high butyrate concentration can inhibit the cell cycle and hinder the repair ability of DNA Double Strand Break (DSB) (Li, 2017), and it is consistent with our results. Butyrate can also change gene expression and prevent cell proliferation by inhibiting the chromatin remodeling activity of histone deacetylases (HDACs) (Koprinarova et al., 2011; Zeng et al., 2020). In our study, we found that *HDAC1* was down-regulated in both 0 mM and 2.5 mM groups, suggesting that its abnormal expression may be associated with nerve cell proliferation disorders (Supplementary Table 11).

The different mechanisms of nerve cell proliferation disorders caused by low and high concentrations of butyrate may explain the contradiction that the abundance of butyrate-producing bacteria in the intestine was positively correlated with the frequency of defecation; however, serum butyrate level is negatively correlated with the frequency of defecation. We speculate that when the abundance of butyric acid-producing bacteria in the intestine reduces, butyrate metabolism decreases, hindering intestinal nerve cell proliferation and slowing down intestinal peristalsis. This may be one of the reasons for the occurrence and initial development of FC. When constipation occurs due to the reduction of butyrate consumption in the intestine, the concentration accumulation increases and inhibits the proliferation of nerve cells, resulting in the continuous impairment of intestinal function, and the absorption of butyrate into the blood also increases correspondingly.

This study has some limitations. First, we did not define the range of butyrate concentration in the intestines of normal people, which needs to be supplemented by subsequent studies. Second, this study was conducted on Chinese women, and thus the results may not be generalized to other populations.

In conclusion, we found that reducing butyric acid-producing bacteria in the gut may cause intestinal nerve cell dysplasia and insufficient energy supply of intestinal cells, resulting in reduced frequency of defecation and increased risk of constipation. Moreover, this showed that high concentrations of butyric acid block the repair of the ENS, making the condition worse. Therefore, maintaining an appropriate concentration of butyrate in the intestine of human constipation patients may also improve the intestinal nervous system, thereby improving constipation. This finding is also a future direction for the treatment of constipation.

## Materials and methods

### Patients and samples preparation

In this study, we collected a total of 530 volunteers from two regions as follows: the experimental and validation groups. Both groups used the same inclusion criteria, sample collection criteria, and sample storage conditions to ensure the reliability of the results. Notably, serum and fecal samples were collected in both groups. Among them, 518 female samples were collected from the Third Affiliated Hospital of Southern Medical University in Guangzhou, Guangdong Province, China, in 2017, and 12 samples were collected from the First Affiliated Hospital of Hunan Medical College in Huaihua, Hunan Province, China, in 2020. This study was approved by the Third Affiliated Hospital of Southern Medical University and was performed in accordance with the principles of the Helsinki Declaration II. The details of the research design have been described in previous studies (Gong et al., 2021; Greenbaum et al., 2022). The inclusion criteria included participants that were: 1. older than 40 years old; 2. who had lived in the sample collection area for more than 3 months.

People with a history of uterus, ovary, and gastrointestinal surgery; serious cardiovascular and cerebrovascular diseases; diabetes; liver disease; gastrointestinal disease; and other diseases related to intestinal microorganisms were excluded from the present study. Each participant signed a written informed consent form before participating in the study. Subsequently, we assessed epidemiological information on age, medical history, family history, physical activity, alcohol use, diet habits, smoking history, defecation frequency, and medical history, among others, using a questionnaire. The Rome IV criteria for functional constipation was used for evaluating gastrointestinal symptoms. Finally, 472 samples with complete information were obtained for follow-up research through screening.

Furthermore, 3 ml of peripheral blood samples were collected from each participant after overnight fasting for at least 8 h, and we isolated serum and extracted genomic DNA from the blood. According to the instruction, feces were collected at the hospital or home and delivered immediately at low temperatures. Fecal samples were obtained for DNA extraction and metagenomic sequencing. According to the manufacturer’s instructions, fecal DNA was extracted using the E.Z.N.A.^®^ Stool DNA Kit (D4015-02, Omega, Inc., USA), and blood DNA was extracted using a SolPure DNA kit (Magen, China). Each sample was frozen at −80^°^C in a freezer.

### Multi-omics data generation and analysis

The fecal samples collected from the experimental (518) and validation (12) groups in this study were sequenced using a shotgun metagenomic sequencing approach, and serum samples were analyzed by gas chromatography-mass spectrometry. The details of the data generation and quality control processes of these samples have been described previously (Lv et al., 2021).

MicrobiomeAnalyst^1^ (Chong et al., 2020) was used to comprehensively analyze the metagenomic data, including diversity analysis, DEseq2, and Linear Discriminant Analysis Effect Size (LEfSe). Finally, we obtained α, β diversity and intergroup differences of intestinal microorganisms. The Kruskal–Wallis test was used for SCFA analysis.

### Isolation and culture of primary mouse intestinal nerve cells

We purchased C57BL/6 mice from Hunan SJA Laboratory Animal Co., Ltd. (Changsha, China), and the 4–6 weeks old C57BL/6 mice were euthanized by intraperitoneal injection of pentobarbital sodium. The intestines were removed and cleaned using Hanks’ balanced salt solution (HBSS; ThermoFisher, USA). After stripping the intestinal mucosa with tweezers, a cell suspension was obtained by digestion and separation with 1 mg/ml collagenase 4 (C4-BIOC, Sigma-Aldrich, Germany). The cells were cultured in Neurobasal A medium specifically designed for neural cells for 48 hours. Each 50 ml of complete medium consists of 47.5 ml Neurobasic A medium (10888022, ThermoFisher, USA), 1 ml B-27 (50×) (17504044, ThermoFisher, USA), 500 μl fetal bovine serum (M3942-500ML, Sigma, USA), 500 μl 200 mM L-glutamine (ThermoFisher, USA), 50 μl glial cell-derived neurotrophin (HY-F0003, MCE, China) and 500 μl antibiotics (100×) (15140122, ThermoFisher, USA). The isolation methods and steps for mouse intestinal primary nerve cells have been previously described (Ye et al., 2020). Animal care and experiments were conducted in accordance with the Guidelines for Animal Experimentation, Central South University, Changsha, Hunan, China, and were approved by the Animal Care and Use Committee of the University.

### Assessment of cell proliferation

Primary nerve cells were plated in 96 well plates with 1,000 cells/well. After culturing with 0.1, 0.5, 1, and 2.5 mM butyrate (B5887, Sigma-Aldrich, Germany) for 72 h, 10 μl Cell Counting Kit-8 (CCK-8, New Cell & Molecular Biotech, China) was added to each well. After 2 hours, absorbance values at 450 nm were examined by a microplate reader (Bio-TEK Instrument, USA).

### Nerve cell immunofluorescence

The cells were fixed with paraformaldehyde, permeabilized with 0.5% Triton X-100 (85111, Thermo, USA), diluted in phosphate-buffered saline (PBS) buffer for 20 min at room temperature, and then blocked with goat serum. Fixed cells were incubated with Tuj-1 (bsm-52323r, BIOSs, China, 1:100 diluted with PBS) at 4^°^C overnight and then incubated with a 1:200 fluorescent dye-labeled secondary antibody (s0001, Affinity, China) at 37^°^C for 1 h. Samples were examined and photographed using a fluorescence microscope (Olympus, Tokyo, Japan). Four photos at different locations were randomly selected, and the cell area was measured using the ImageJ software.

### RNA-seq and pathway enrichment analysis

Total RNA was extracted from mouse intestinal nerve cells using TRIzol reagent (15596018, Invitrogen, USA), and each group was prepared with three parallel replicates. All the RNA samples were sent to BGI Corporation (Shenzhen, China) for RNA-seq analysis via a BGISEQ-500 sequencer. They were averagely generating about 1.19 Gb bases per sample. The average mapping ratio with reference genome was 93.99%; 18,514 genes were identified. The pathway analysis for DEGs was performed based on the KEGG database. The data were analyzed on the Dr. Tom network platform of BGI.

### Statistical analysis

Differences between two or more groups were analyzed using the independent samples *t*-test. When variances were not homogeneous, the data were analyzed using the Kruskal–Wallis test. All tests were performed using the SPSS 21 software. The correlation analysis of defecation frequency, serum butyric acid concentration, bacterial OTUs relative abundance, and the metabolic pathway was evaluated using the Spearman correlation coefficient. Statistical significance was set at *P* < 0.05.

## Data availability statement

The datasets presented in this study can be found in online repositories. The names of the repository/repositories and accession number(s) can be found below: BioProject, PRJNA950204.

## Ethics statement

This study was approved by the Third Affiliated Hospital of Southern Medical University and was performed in accordance with the principles of the Helsinki Declaration II. The patients/participants provided their written informed consent to participate in this study. This animal study was reviewed and approved by Animal Care and experiments were conducted in accordance with the Guidelines for Animal Experimentation, Central South University, Changsha, Hunan, China, and were approved by the Animal Care and Use Committee of the university.

## Author contributions

LW, W-QL, XL, H-JT, R-PQ, and P-PL contributed to the data curation. LW, W-QL, and H-ML participated in the formal analysis. H-WD and H-MX contributed to the project conception, design, and initiation. LW and J-TY participated in the design and performance of the *in vitro* experiments. JS, H-WD, and H-MX supervised the study. LW contributed in the writing of the original draft. LW, XL, HS, H-WD, and H-MX participated in the writing of the review and editing. All authors contributed to the article and approved the submitted version.

## Footnotes

1
https://www.microbiomeanalyst.ca
