# Supplementary material for: Enteric nervous system damage caused by abnormal intestinal butyrate metabolism may lead to functional constipation
Source: Front Microbiol. 2023 May 9;14:1117905. doi: 10.3389/fmicb.2023.1117905 (PMC10203953; doi:10.3389/fmicb.2023.1117905)
Supplement: Supplementary file 1 [file Data_Sheet_1.docx]

**Supplementary materials**

**
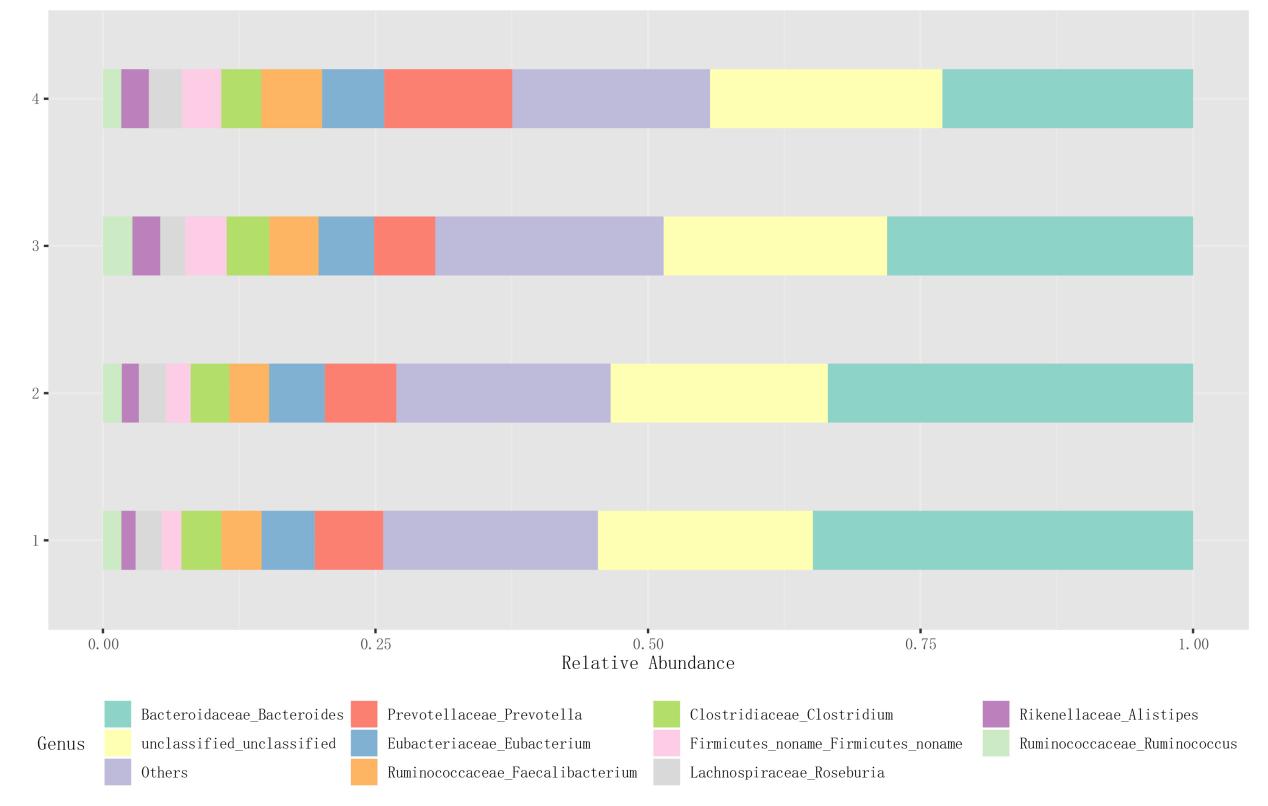
**

**Supplemental Figure 1.** The shift of gut microbiota in the four groups according to the metagenomic data.Metagenomic sequencing was performed for all four groups. The compositions and relative abundances of intestinal microorganisms in the four groups were different at the genus level.


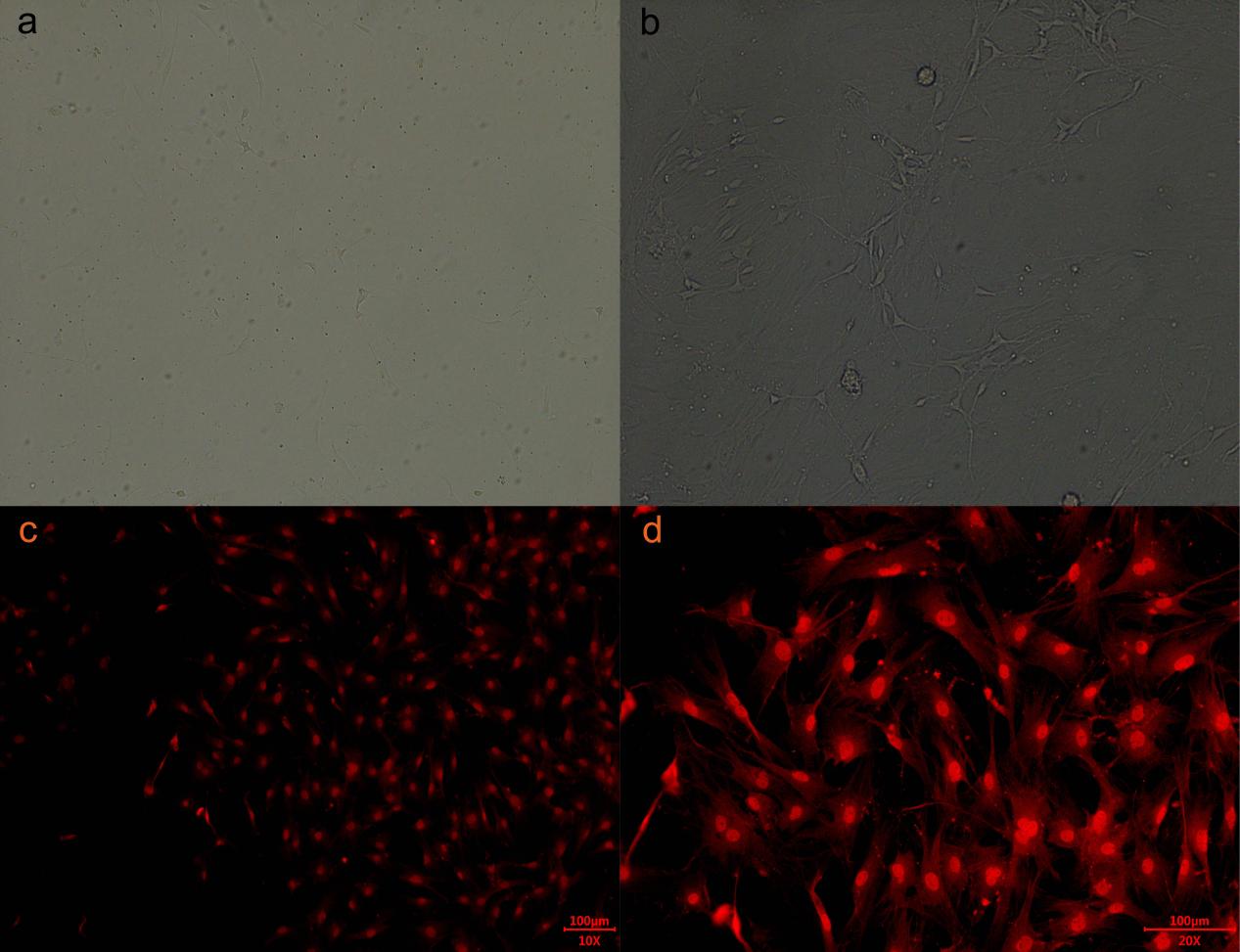


**Supplemental Figure 2**. Identification of isolated nerve cells with Tuj-1 antibody.

a,b:10x and 20x cells，c,d:10x and 20x Tuj-1 immunofluorescence

**
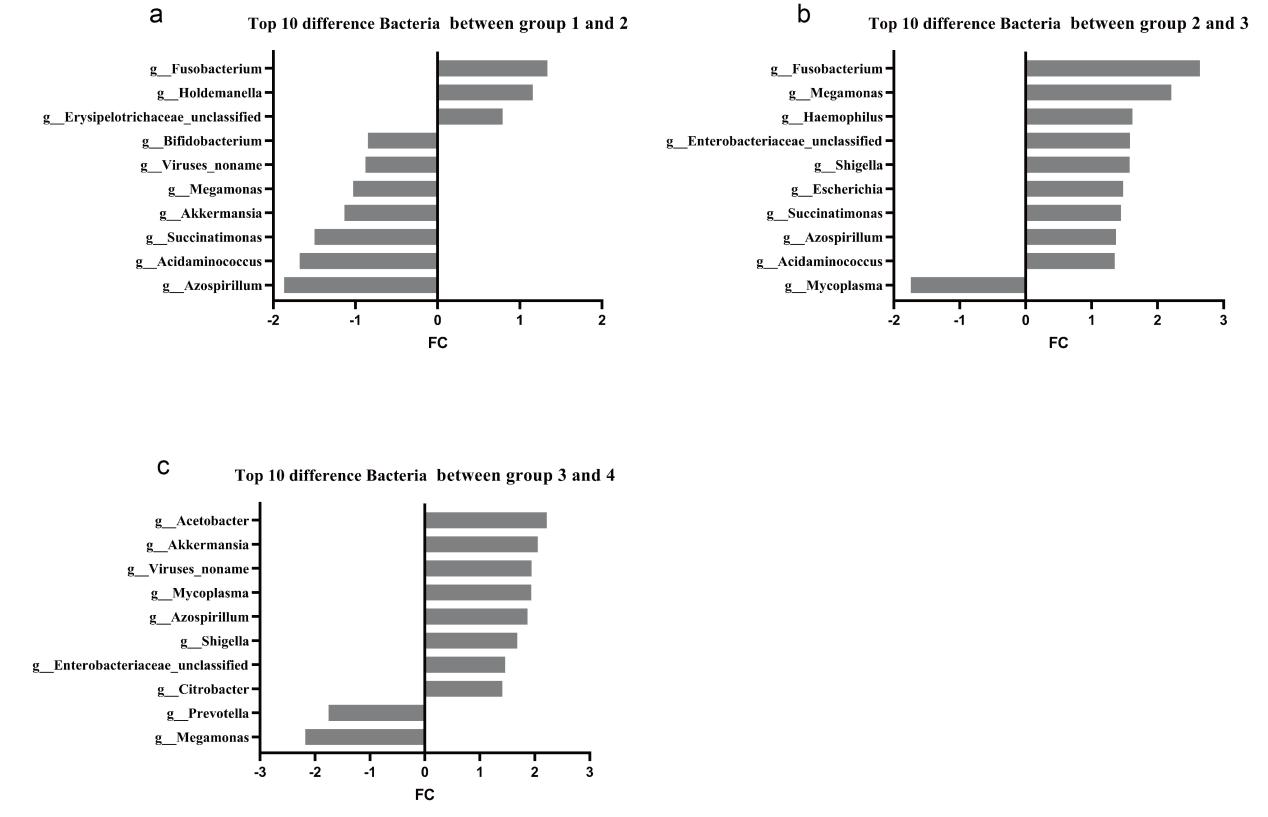
**

**Supplemental Figure 3.** The top 10 difference bacterias among the four groups obtained by Deseq2, FC=log^2^ fold change at the genus levels .


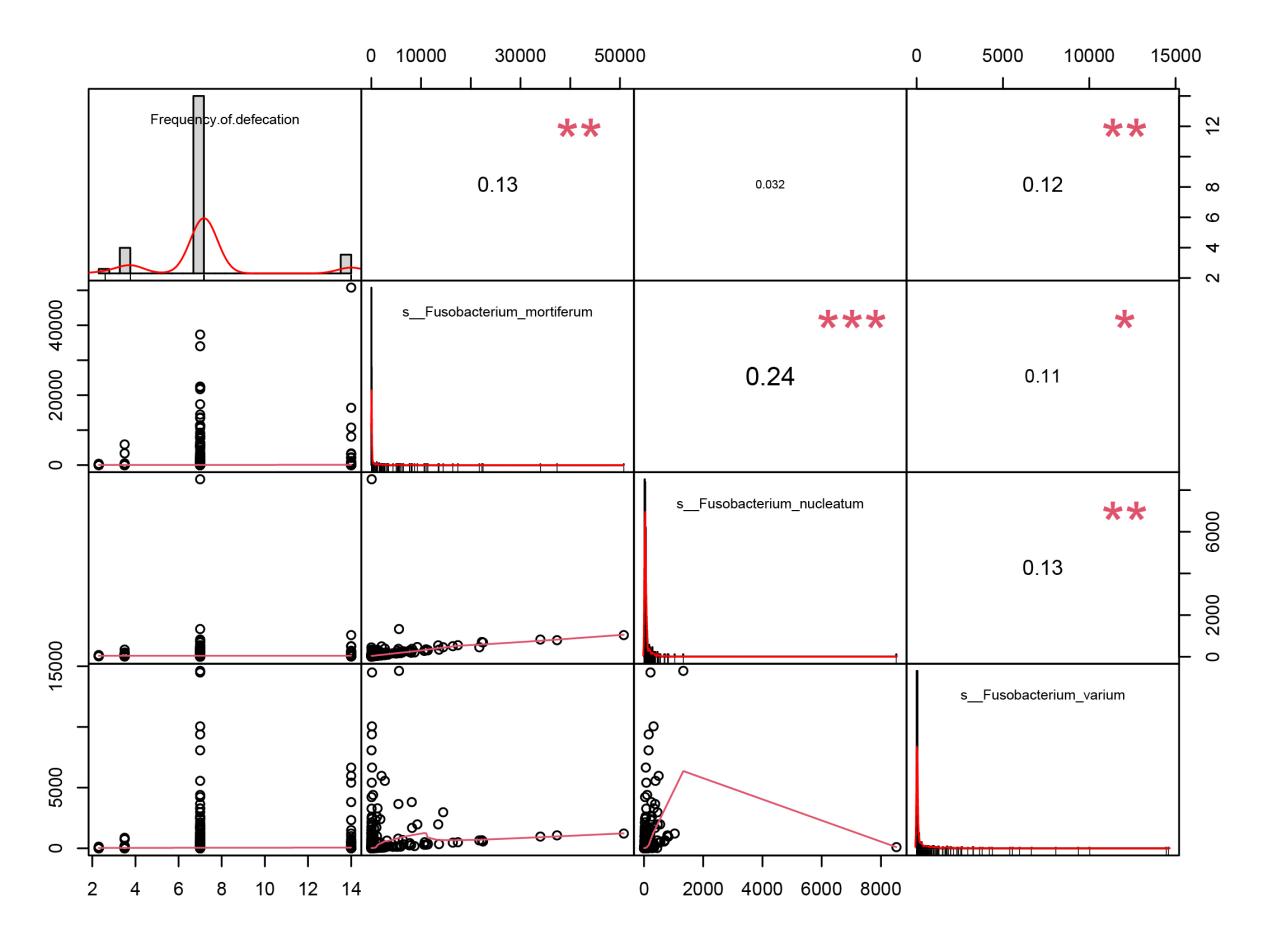


**Supplemental Figure 4.** Correlation analysis of *s_Fusobacterium_ulcerans*, *s_Fusobacterium_varium*, *s_Fusobacterium_mortiferum* and defecation frequency in the population.

**
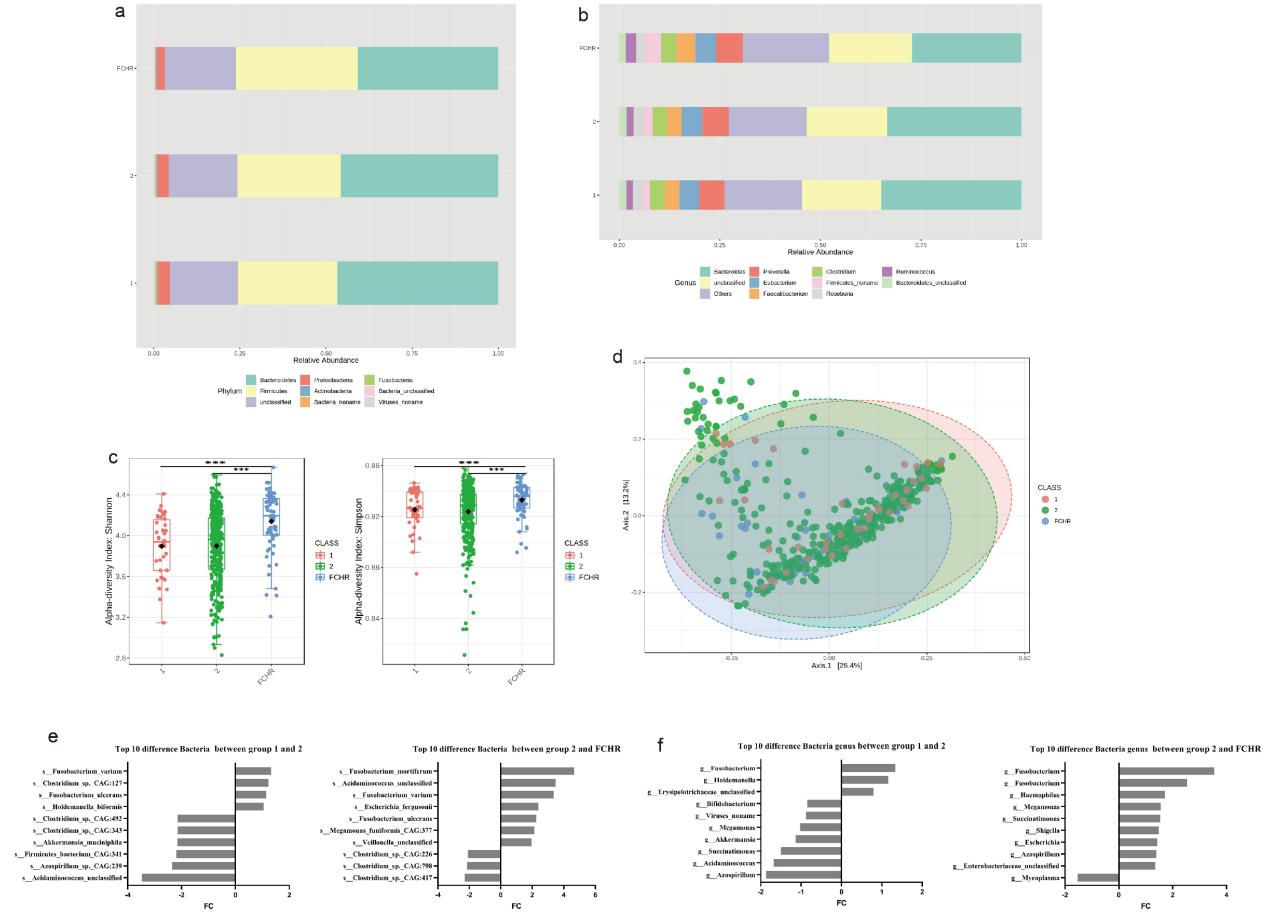
**

**Supplementary Figure 5.** The shift of intestine microbiota in the three groups according to the metagenomic data.Metagenomic sequencing was performed for all group 1, 2 and FCHR. (a, b) The compositions and relative abundances of intestinal microorganisms in the three groups were different at the phylum and genus levels. (c) Shannon and Simpson diversity in three groups. Shannon : P=0.0002 for the comparison between group 1 and group FCHR, P=1E-10 for the comparison between group 2 and group FCHR. Simpson ： P=0.015 for the comparison between group 1 and group 3, P=8E-06 for the comparison between group 1 and group FCHR. (d) Principal coordinate analysis, P=0.005 for the comparison between group 1 and group FCHR, P=0.001 for the comparison between group 2 and group FCHR. (e) The top 10 difference bacterias among the three groups obtained by Deseq2, FC=log^2^ fold change at the genus and species levels .

**
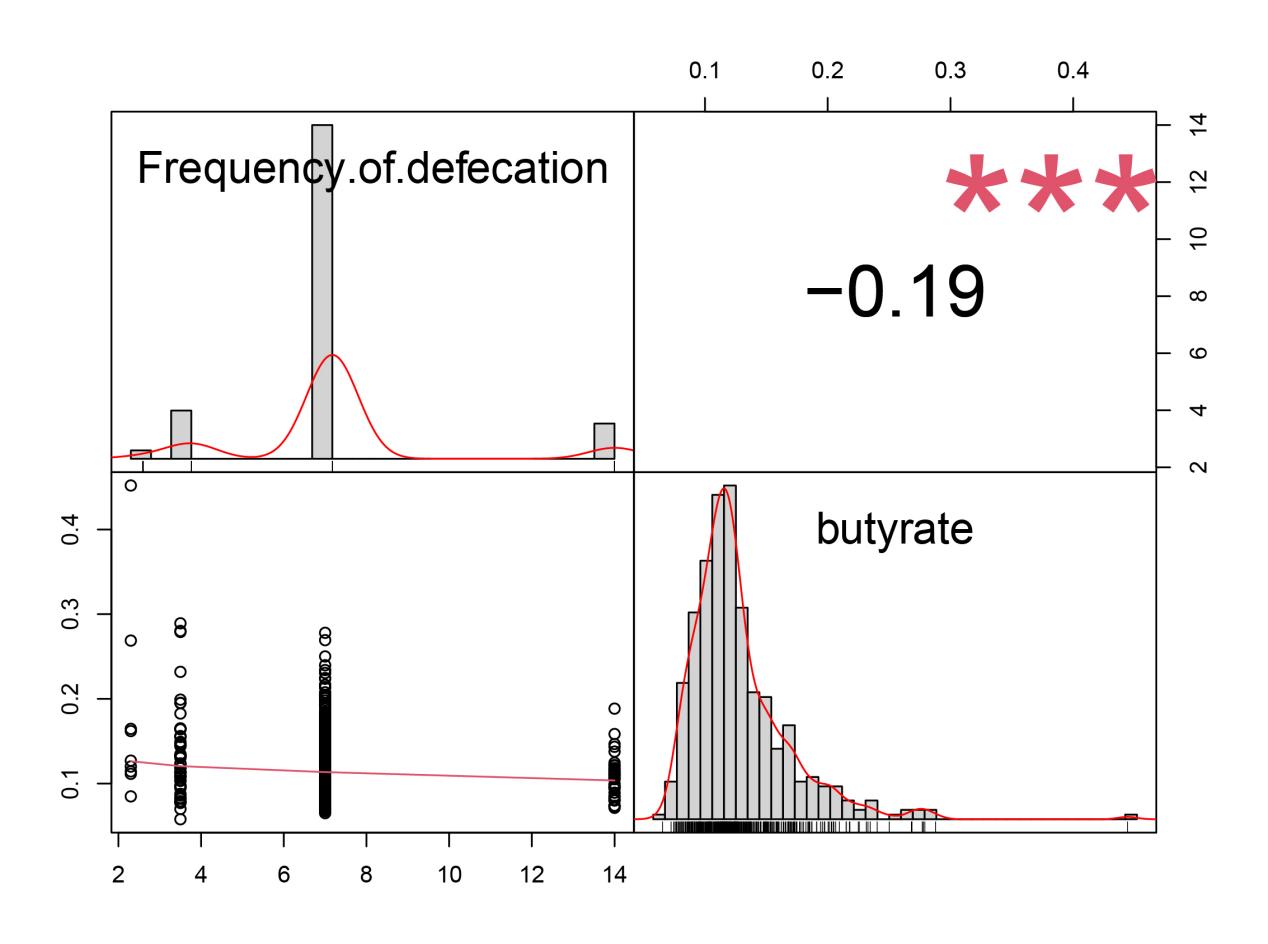
**

**Supplementary Figure 6.** Correlation analysis of butyrate and frequency of defecation in the population.


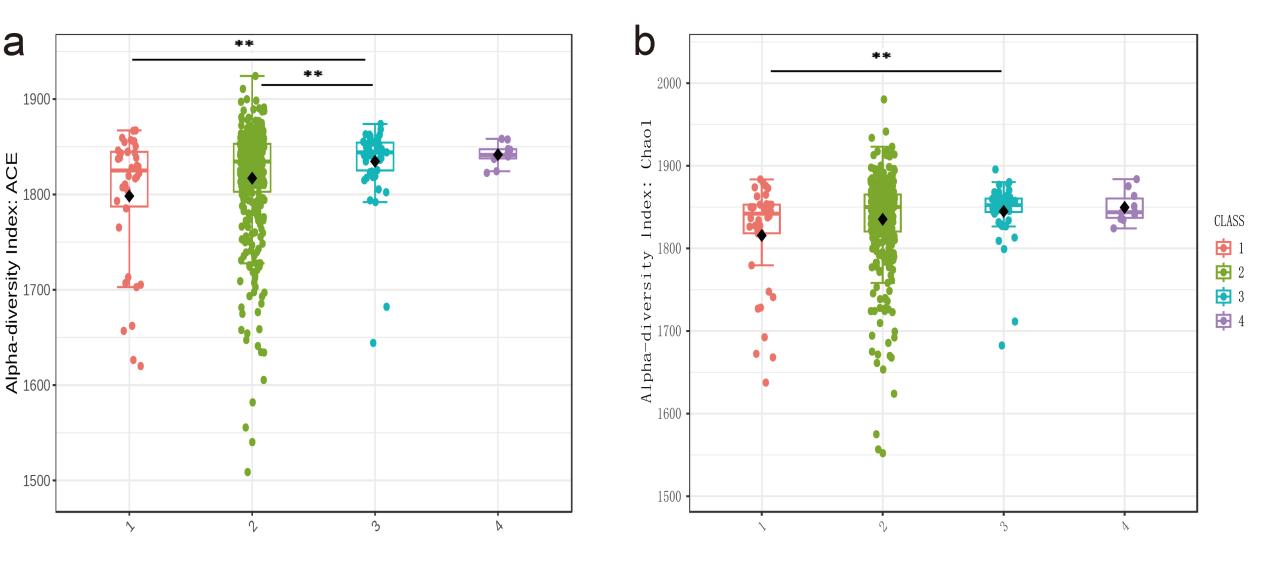


**Supplementary Figure 7.** Chao1 and ACE diversity in four groups. (a) ACE diversity in four groups. P=0.015 for the comparison between groups 1 and 3, P=0.006 for the comparison between groups 2 and 3. (b) Chao1 diversity in four groups. P=0.01 for the comparison between groups 1 and 3.

**Supplemental Table 1.** Questionnaire information of 460 postmenopausal women and 12 premenopausal women. The main contents include： age, diet, defecation status, mental status and other information.

**Supplemental Table 2.** This table includes operational genomic unit (OTU) data of 472 individuals' metagenomes.To screen out the intestinal microorganisms that play an important role in the intestine, we selected the microbial species whose abundance was more than 0.01% of the overall data, the number after screening was 625 in 460 postmenopausal women, the number after screening was 406 in 12 premenopausal women，

**Supplemental Table 3.** The data in this table include the concentration of targeted SCFAs in serum of 472 samples, including four main SCFAs: butyric acid, acetic acid, valeric acid, propionic acid.

**Supplemental Table 4.** To clarify the correlation between the type of bacteria and defecation frequency, we analyzed the intestinal bacterial composition of 460 samples by deseq2. We found that there were significant differences in *Fusobacterium varium* and *Fusobacterium mortiferum* in groups 1, 2, 3 and the difference was positively correlated with defecation frequency in 4 groups. *Fusobacterium varium* and *Fusobacterium mortiferum* ranks in the top 10 in the table, indicating that it may be a key difference bacterium.

**Supplemental Table 5.** To clarify the correlation between the type of bacteria and defecation frequency, we analyzed the intestinal bacterial composition of 12 samples by LEFSe. We found that *Fusobacterium Ulcerans* is the most significant strain in the two groups, while *Fusobacterium Mortiferum* is also in the forefront.

**Supplemental Table 6.** This table includes operational genomic unit (OTU) data of 472 individuals' metagenomes.There were no significant difference between butyrate producing bacteria and methanogens such as F*aecalibacterium_prausnitzii*, *butyrateimonas_virosa*, *g__Lactobacillus*, *g__Bifidobacterium*， methanogenic genus Gelria in the population.

**Supplemental Table 7.** The KEGG module differential metabolic pathway analysis based on level 2.

**Supplemental Table 8.** We performed GO,KEGG and GSEA enrichment analysis on these differentially expressed genes.Compared with the 0.5mM group,it was found that 21 pathways were up-regulated, 48 pathways were down regulated in the 0mM group, 41 pathways were up regulated and 4 pathways were down regulated in the 2.5mM group.

**Supplemental Table 9 Data of cell proliferation detected by CCK8**

|  | **N** | **Minimum（OD450）** | **Maximum（OD450）** | **Average** |
| --- | --- | --- | --- | --- |
| 0 mM | 3 | 215.33 | 217 | 215.89±0.96 |
| 0.1 mM | 3 | 215 | 217 | 215.89±0.96 |
| 0.5 mM | 3 | 242 | 246.5 | 243.5±2.6 |
| 1 mM | 3 | 215.67 | 220.67 | 217.33±2.89 |
| 2.5 mM | 3 | 206 | 213.33 | 208.44±4.23 |

**Supplemental Table 10 Cell area in 4 randomly selected visual fields under microscope**

|  | **N** | **Minimum cellarea** | **Maximum cell area** | **Average** |
| --- | --- | --- | --- | --- |
| 0 mM | 99 | 2839 | 27150 | 9639.47±4943.49 |
| 0.1 mM | 91 | 2247 | 27701 | 10492.76±5687.76 |
| 0.5 mM | 101 | 4406 | 32612 | 12619.54±5713.02 |
| 1 mM | 98 | 38560 | 28592 | 9945.74±5361.02 |
| 2.5 mM | 100 | 2241 | 19630 | 6307.05±3398.75 |

**Supplemental Table 11 Expression of AHR and HDAC1 in three groups**

|  |  | **N** | **Minimum** | **Maximum** | **Average** |
| --- | --- | --- | --- | --- | --- |
| AHR | 0 mM | 3 | 4.33 | 6.66 | 5.58±1.17 |
|  | 0.5 mM | 3 | 12.58 | 17.01 | 14.12±2.50 |
|  | 2.5 mM | 3 | 9.51 | 24.18 | 14.45±8.43 |
| HDAC1 | 0 mM | 3 | 146.7 | 163.09 | 156.97±8.95 |
|  | 0.5 mM | 3 | 199.75 | 213.01 | 205.54±6.79 |
|  | 2.5 mM | 3 | 170.19 | 224.86 | 189.01±31.06 |

**Study design overview**

In this study, a total of 518 perimenopausal and postmenopausal female volunteers were recruited in the Third Affiliated Hospital of Southern Medical University, Guangzhou in November 2017 according to the inclusion and exclusion criteria. We collected peripheral blood and fecal samples from these volunteers, performed metagenomic sequencing and targeted detection of serum SCFAs. After excluding any individuals with missing data on one of the multi-omics data, we obtained multi-omics data from 460 Chinese Han menopausal women for the subsequent analysis. The subjects were divided into 4 groups. Referring to Rome IV for the diagnostic of FC, group 4 was identified as FC patients, group 3 could be considered as high-risk people for FC, and groups 1 and 2 were low-risk people. To further validate our findings, we recruited an independent sample of 12 premenopausal women, including 6 patients with FC and 6 healthy controls (HC), in Huai Hua City, Hunan Province, China. Finally, in order to explore the effect and mechanism of butyric acid on the development of intestinal nerve cells, we cultured mouse intestinal nerve cells *in vitro* with different concentrations of butyric acid.

**\
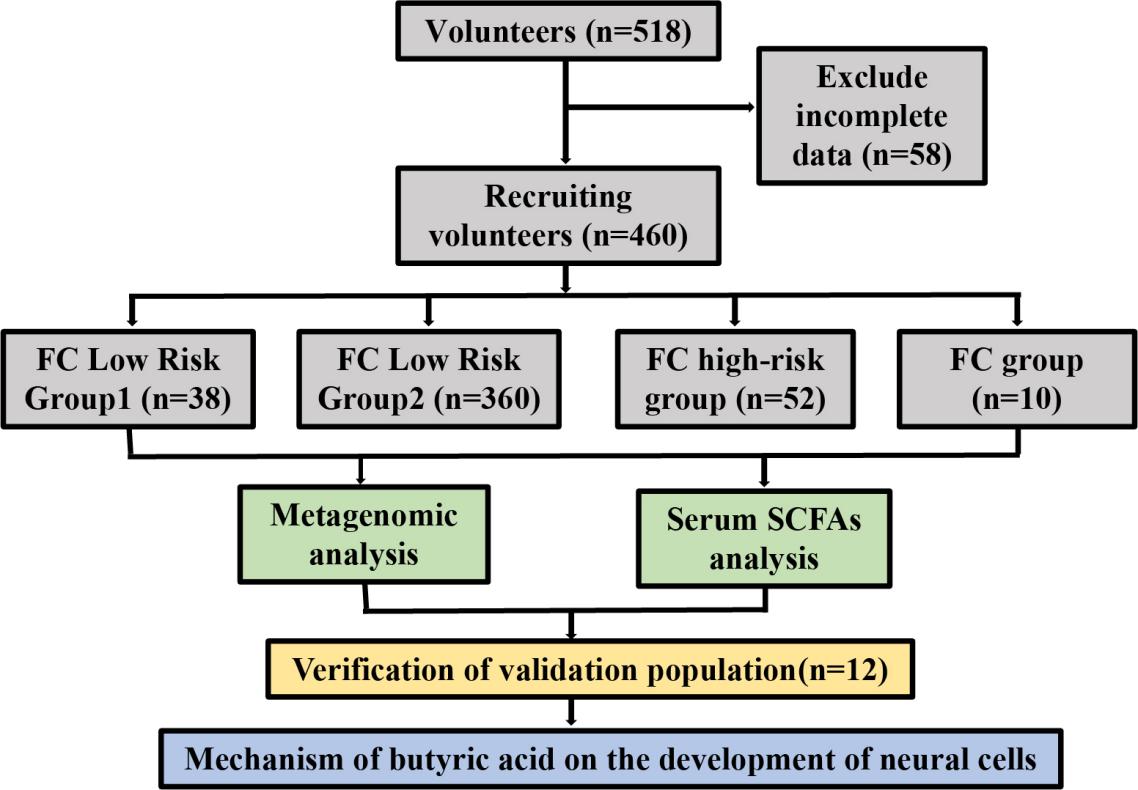
**

Supplementary Figure 8. Study design overview

**Total RNA extraction**

Total RNA was extracted from the tissues using Trizol (Invitrogen, Carlsbad, CA, USA) according to manual instruction. About 60 mg of tissues were ground into powder by liquid nitrogen in a 2 mL tube, followed by being homogenized for 2 minutes and rested horizontally for 5 minutes. The mix was centrifuged for 5 minutes at 12,000×g at 4°C, then the supernatant was transferred into a new EP tube with 0.3 mL chloroform/isoamyl alcohol (24:1). The mix was shacked vigorously for 15s, and then centrifuged at 12,000×g for 10 minutes at 4°C. After centrifugation, the upper aqueous phase where RNA remained was transferred into a new tube with equal volume of supernatant of isopropyl alcohol, then centrifuged at 13,600 rpm for 20 minutes at 4°C. After deserting the supernatant, the RNA pellet was washed twice with 1 mL 75% ethanol, then the mix was centrifuged at 13,600 rpm for 3 minutes at 4°C to collect residual ethanol, followed by the pellet air dry for 10 minutes in the biosafety cabinet. Finally, 100µL of DEPC-treated water was added to dissolve the RNA. Subsequently, total RNA was qualified and quantified using a Nano Drop and Agilent 2100 bioanalyzer (Thermo Fisher Scientific, MA, USA).

**mRNA Library Construction**

Oligo(dT)-attached magnetic beads were used to purified mRNA. Purified mRNA was fragmented into small pieces with fragment buffer at appropriate temperature. Then First-strand cDNA was generated using random hexamer-primed reverse transcription, followed by a second-strand cDNA synthesis. afterwards, A-Tailing Mix and RNA Index Adapters were added by incubating to end repair. The cDNA fragments obtained from previous step were amplified by PCR, and products were purified by Ampure XP Beads, then dissolved in EB solution. The product was validated on the Agilent Technologies 2100 bioanalyzer for quality control. The double stranded PCR products from previous step were heated denatured and circularized by the splint oligo sequence to get the final library. The single strand circle DNA (ssCir DNA) was formatted as the final library. The final library was amplified with phi29 to make DNA nanoball (DNB) which had more than 300 copies of one molecular, DNBs were loaded into the patterned nanoarray and single end 50 bases reads were generated on BGIseq500 platform (BGI-Shenzhen, China).

**Data Analysis**

The sequencing data was filtered with SOAPnuke (v1.5.2)[1] by (1) Removing reads containing sequencing adapter; (2) Removing reads whose low-quality base ratio (base quality less than or equal to 5) is more than 20%; (3) Removing reads whose unknown base ('N' base) ratio is more than 5%, afterwards clean reads were obtained and stored in FASTQ format. The clean reads were mapped to the reference genome using HISAT2 (v2.0.4)[2]. Bowtie2 (v2.2.5)[3] was applied to align the clean reads to the reference coding gene set, then expression level of gene was calculated by RSEM (v1.2.12)[4]. The heatmap was drawn by pheatmap (v1.0.8)[5] according to the gene expression in different samples. Essentially, differential expression analysis was performed using the DESeq2 (v1.4.5)[6] with Q value ≤ 0.05. To take insight to the change of phenotype, GO (http://www.geneontology.org/) and KEGG (https://www.kegg.jp/) enrichment analysis of annotated different expressed gene was performed by Phyper (https://en.wikipedia.org/wiki/Hypergeometric_distribution) based on Hypergeometric test. The significant levels of terms and pathways were corrected by Q value with a rigorous threshold (Q value ≤ 0.05) by Bonferroni[7]. Quality control of RNA-seq data, including correlation analysis, box plot analysis are included in [Supplementary Figure 7.](https://www.frontiersin.org/articles/10.3389/fnmol.2019.00332/full#TS1) and [Supplementary Figure 8.](https://www.frontiersin.org/articles/10.3389/fnmol.2019.00332/full#DS1)


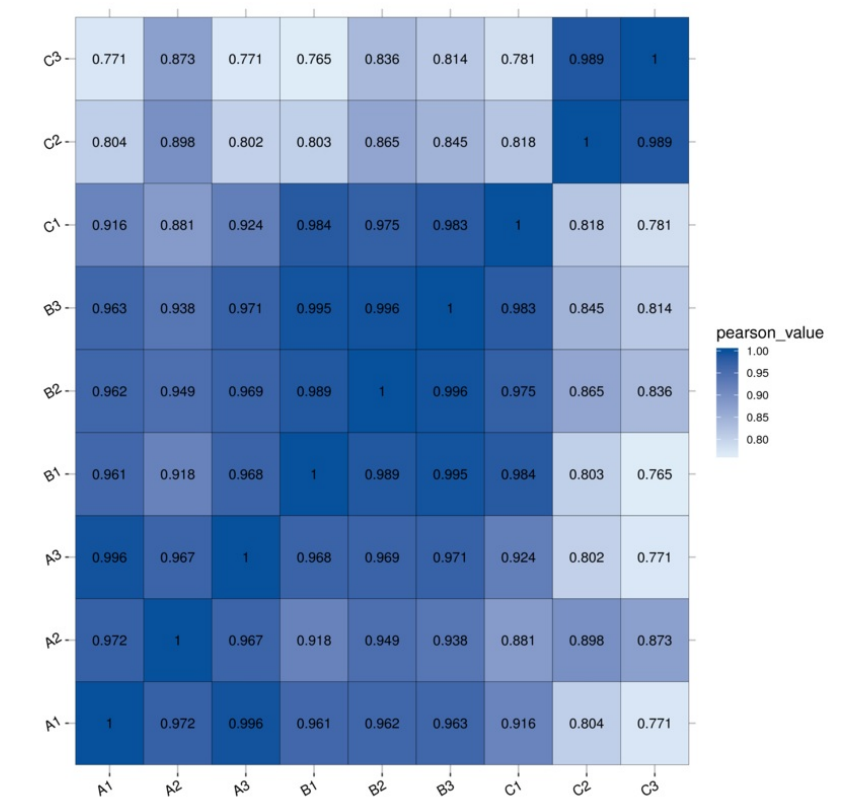


Supplementary Figure 9. In order to reflect the correlation of gene expression between samples, the Pearson correlation coefficients of all gene expressions between each two samples were calculated, and these coefficients were reflected in the form of a heatmap. The correlation coefficients can reflect the similar situation of the overall gene expression between each sample. The higher the correlation coefficient is, the more similar the gene expression level is.


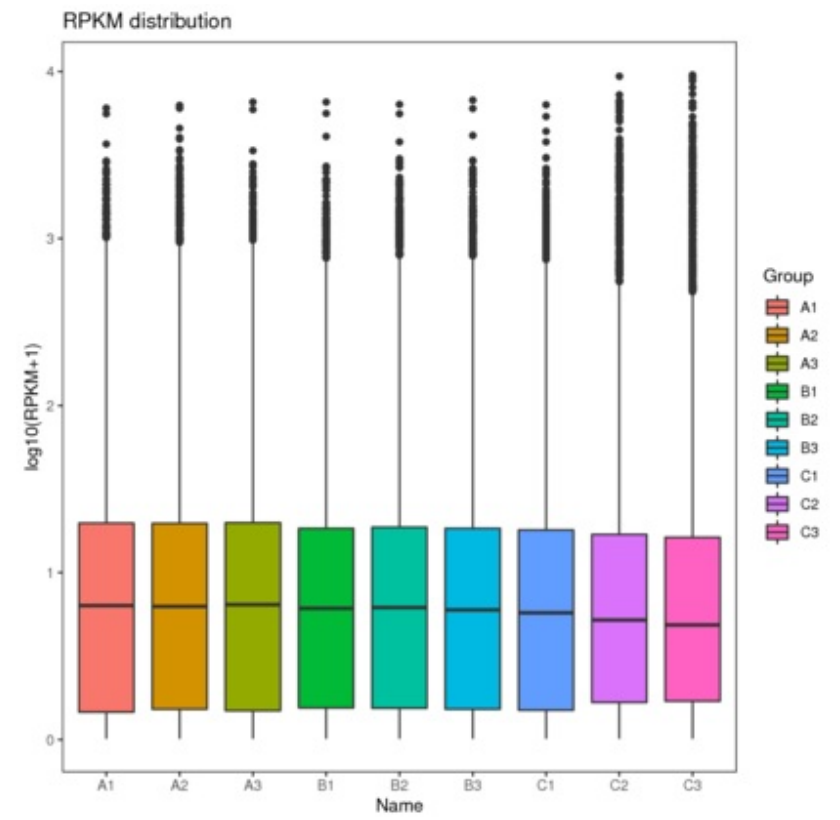


Supplementary Figure 10. The boxplot show the distribution of gene expression levels of each sample.

References:

[1] Li R, Li Y, Kristiansen K, Wang J. (2008). SOAP: short oligonucleotide alignment program. Bioinformatics. 24(5):713-4.

[2] Kim, D., Langmead, B. & Salzberg, S. L. HISAT: a fast spliced aligner with low memory requirements. Nat. Methods 12, 357-360 (2015).

[3]Langmead, B. et al. Fast gapped-read alignment with Bowtie 2. Nat. Methods 9, 357-359 (2012).

[4] Li, B. & Dewey, C. N. RSEM: accurate transcript quantification from RNA-Seq data with or without a reference genome. BMC Bioinformatics 12, 323 (2011).

[5] Raivo Kolde. Package ‘pheatmap’. 2019-01-04 13:50:12 UTC.

[6] Love, M. I., Huber, W. & Anders, S. Moderated estimation of fold change and dispersion for RNA-seq data with DESeq2. Genome Biol. 15, 550 (2014).

[7] Abdi, H. The Bonferonni and Sˇidák Corrections for Multiple Comparisons. Encycl

Meas Stat. 2007; 1: 1–9.
